# The Effect of Extra Educational Elements on the Confidence of Undergraduate Dental Students Learning to Administer Local Anaesthesia

**DOI:** 10.3390/dj9070077

**Published:** 2021-07-01

**Authors:** Mats Sjöström, Malin Brundin

**Affiliations:** 1 Department of Oral and Maxillofacial Surgery, Umeå University, 90187 Umeå, Sweden; mats.sjostrom@umu.se; 2 Department of Endodontics, Umeå University, 90187 Umeå, Sweden

**Keywords:** undergraduate dental education, clinical skills teaching, teaching methodology, local anesthesia

## Abstract

Local anaesthesia is taught early in the practical part of dental programs. However, dental students express uncertainty and concern before their practical training in local anaesthesia. The aim of this study was to evaluate how extra educational elements in the teaching of local anaesthesia affect students’ confidence using local anaesthesia. The students were divided into three groups (A, B and C). Group A received the same education that was used the previous year (i.e., four hours of theoretical lectures followed by four hours of practical exercises performed on a fellow student). Group B did their practical training on fellow students in groups of three, with each student taking turns performing, receiving and observing the procedure. Group C received training using an anatomically correct model before their practical training on a fellow student. After each training step, the students completed a questionnaire about their confidence administering local anaesthesia. The students experienced a significant increase in confidence after each educational step. Combining theory and practical instruction, including the use of anatomically correct models and peer instruction, improved students’ confidence in administering local anaesthesia. The greatest increase in confidence was in the students placed in groups of three where each student performed, received and observed the procedure.

## 1. Introduction

Dental education involves clinical training on patients. This implies a need for student teaching of local anesthesia for patient comfort. A recently published review of teaching local anaesthesia in dental schools in the USA identified three main pedagogical approaches: didactic instruction via books and lectures; practical exercises conducted in peer groups; and practice using anatomically correct models. However, due to the heterogeneity of the studies, the authors were unable to analyse the pedagogical effects of the three teaching methods and the students’ confidence administering local anaesthesia. The authors concluded that the lack of evidence makes it difficult to determine an optimal way to teach local anaesthesia [1].

Dental professionals can feel anxious about local anaesthesia, resulting in stress and anxiety before administering local anaesthesia to a patient. The amount of practice affects stress and anxiety levels, where less practice or experience increases stress and anxiety. Stress and reasons for stress have been evaluated in previous studies. For example, Wong et al. studied dental hygiene students’ experiences of anxiety related to their confidence performing local anaesthesia. An increased degree of confidence reduced students’ stress associated with administering local anaesthesia [2]. In a paediatric anaesthesia study, a higher degree of stress was found in dental students compared with experienced specialist colleagues [3]. Methods other than direct clinical training have been found to be effective and improve students’ confidence administering local anaesthesia. For example, Kenny et al. found that students trained in administering local anaesthesia to children using videos showed significantly increased confidence immediately after the training as well as at follow-up [4]. The use of anatomically correct models designed to verify whether local anaesthesia is correctly administered is a teaching method that can eventually become a complement to the use of peers for practice. Moreover, training with these models provides an opportunity to train an unlimited number of times and without continuous supervision. Previous evaluations of preclinical model training have concluded that students who practiced on a model before their clinical training were significantly better prepared and more confident administering local anaesthesia to a peer [5]. In addition, training on a model has been associated with increased confidence and better motor control [6]. However, model training does not automatically increase a dental student’s ability to safely administer local anaesthesia to a patient [7]. Accessibility to reflection is an important part of the learning process according to Kolb’s learning cycle [8]. The time and opportunities for this should be included in practical training elements. This can be done by ensuring that there is time for students to observe clinical situations without them being the one performing the procedure.

Dental students in Umeå are taught local anaesthesia as one of the first clinical topics, early in the fifth semester. The teaching curriculum includes literature studies, lectures, demonstrations, focus groups and treatment of patients. The practical training of local anaesthesia is carried out on a fellow student after demonstration followed by personal clinical training under the supervision of an oral surgeon. As a final element of the course, the student administers local anaesthesia under supervision to a patient prior to tooth extraction. The stepwise education of local anaesthesia, with clinical observations during instruction and demonstrations of how to administer local anaesthesia performed by the oral surgeon, followed by personal training under supervision, can be compared to a master teaching a novice or a novice studying under a master. Solid knowledge grounded in theory is important to achieve excellence in the students’ practice. Active learning by using instructional activities and involvement with the students who are being trained in local anaestheisia gives the students space to reflect on the clinical situation and their own performance. This reflective thought and an internal processing links the experience with previous learning [9]. The student evaluations collected after each completed course report that the students express a need for more clinical training to increase their confidence when administering local anaesthesia to patients.

This study aimed to evaluate three different teaching models, including one that utilized anatomical models in addition to clinical observations, during training of dental students in local anaesthesia at Umeå University. Hopefully, the results can improve the teaching of local anaesthesia to dental students in Umeå University and increase the students’ confidence during application of local anaesthesia.

## 2. Materials and Methods

In 2020, all undergraduate dental students at Umeå University in their fifth semester (*n* = 72, 50 women/22 men, mean age: 25, range 21–39) were invited to a study evaluating whether extra educational elements can improve student confidence for local anaesthesia. The students were informed orally and in writing about the study and that their participation was voluntary. After acceptance, all students were given a survey consisting of a statement to be answered with a classification for confidence on a visual analogue scale ranging between zero and ten (0 = I feel absolutely unconfident in independently administering anaesthesia to patients and 10 = I feel completely confident in independently administering anaesthesia to patients) [10,11] (Appendix A).

**Figure 1:**
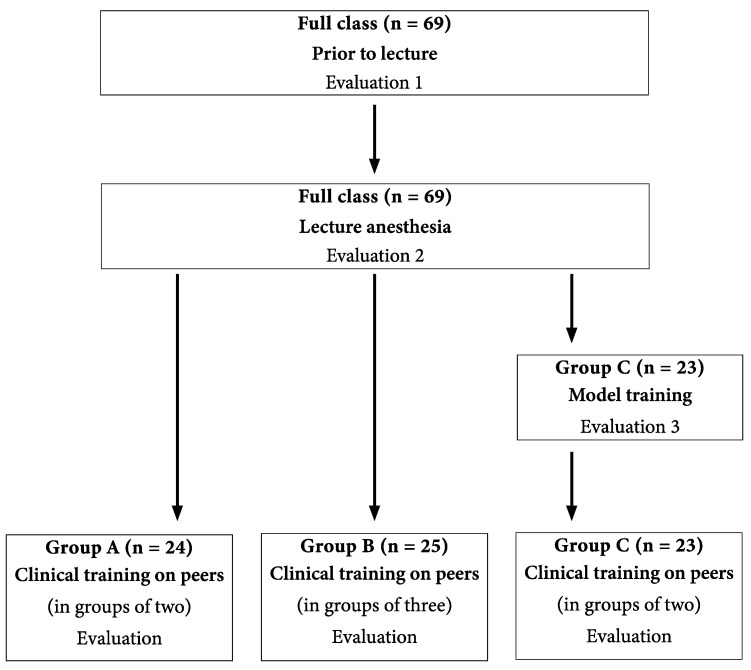
Study design for evaluation of students’ self confidence in using local anesthesia during the course “Oral surgery 1”, fifth semester. Three students were not present at the theoretical lecture, which is why their answers are missing in evaluations 1 and 2. At the clinical training, all 72 students participated.

The students were divided into three groups (A, B and C) during their dental program. One group received the traditional education while two groups received modified education. All groups received training in accordance with the syllabus. A randomization between the groups was performed, resulting in group A (*n* = 24, 14 women/10 men, mean age: 25, range 21–31) receiving the traditional education of practical training in local anaesthesia—i.e., in groups of two, with a four-hour theory lecture followed by four hours of practical training on a fellow student. Group B (*n* = 25, 19 women/6 men, mean age: 25, range 22–39) received a four-hour theory lecture followed by four hours of practical training in groups of three—i.e., each member of the group performed, received and observed the procedure. Group C (*n* = 23, 17 women/6 men, mean age: 25, range 22–36) received a four-hour theory lecture followed by four hours of practical training on an anatomically correct model (Frasaco, AG-3 IB) (Figure 1).

**Figure 2:**
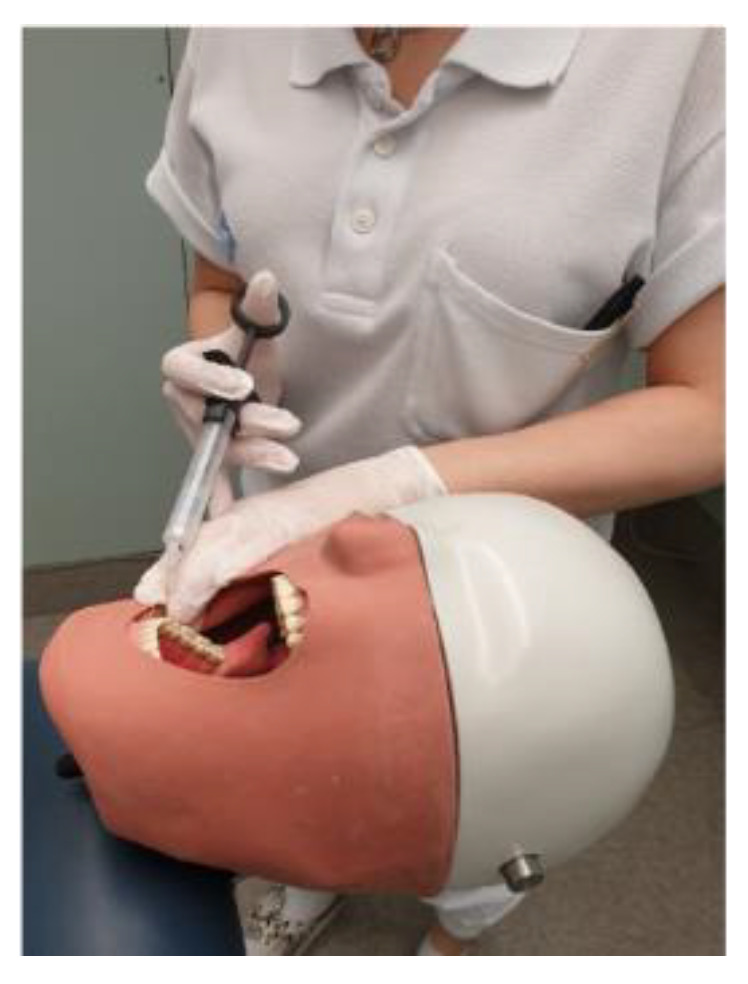
Dental student during model training.

The model has four contact points (mental foramen, mandibular foramen, incisive foramen and greater palatine foramen) covered by rubber simulating covering mucosa. The students simulated injections with dry injection needles and the right positioning of the needle tip was indicated with acoustic signals (Figure 2).

After model training, group C had four hours of practical training on a fellow student (i.e., a group of two). As group C was uneven, one sub-group consisted of three students instead of two. All students (groups A, B and C) were taught the inferior alveolar block technique using the direct standard technique. During all the local anaesthesia attempts, four oral surgeons supervised the groups. After each training step, the students completed the survey described above. The responses were compiled on two occasions and the mean of the two measured values was used in the statistical calculations.

Parts of the course (the clinical training) were performed during the COVID-19 pandemic. At the end of March, all teaching at universities in Sweden was conducted digitally. Exceptions were made, however, for education where clinical elements were included. Clinical training was allowed to be performed under special restrictions, including full protective equipment and adaptation of training facilities. The clinical education at the School of Dentistry in Umeå thus continued during the pandemic. The introductory lecture in this case could be held on the university’s premises in the traditional way, as this preceded in time the transition to digital education.

Statistics

**Figure 3:**
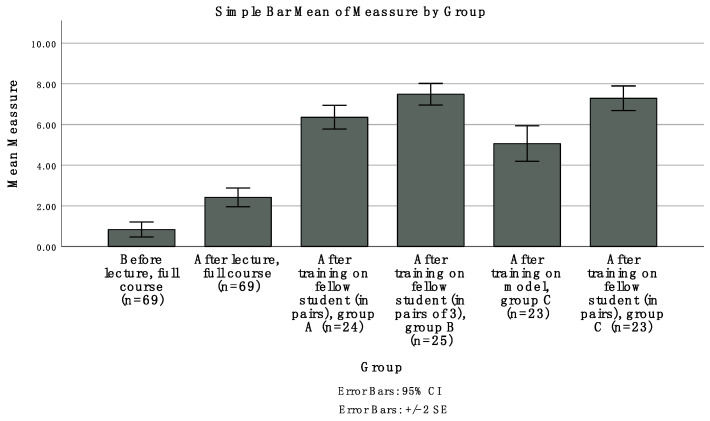
Students’ self-evaluation about self-confidence in independently administering anesthesia to patients before the theoretical lecture, after the theoretical lecture, after training on a model and after training on a fellow student. Each question was answered based on self-assessment on a 10-point graded scale (0 = absolutely not confident in independently administering anesthesia to patients, 10 = completely confident in independently administering anesthesia to patients).

The non-parametric Mann–Whitney U-test was used to compare the students’ self-evaluated measures of perceived safety between the three groups. For all tests, the *p*-value was set to ≤0.05. The responses to the survey are presented as mean values and the corresponding standard error of the mean for each occasion (Figure 3). All statistical tests were performed using IBM SPSS Statistics for Windows, Version 26.0 (IBM Corp: Armonk, NY, USA).

## 3. Results

The results of the students’ self-evaluation for confidence after each educational step are presented in Figure 3. Three students were not present at the theoretical lecture, which is why their answers are missing in evaluations 1 and 2. At the clinical training all 72 students participated.

Table 1 describes the statistical analysis of differences between the educational steps as expressed in students’ confidence administering local anaesthesia. A significant increase in confidence administering local anaesthesia was seen after the theoretical lecture (*p*-value < 0.01). Significant differences in confidence administering local anaesthesia were also seen between the theoretical lecture and the students training on each other (i.e., peer training under supervision of an oral surgeon) (*p*-value < 0.01). A non-significant difference was seen between group C (training with the model followed by peer training in pairs) and group B (peer training in groups of three; *p*-value 0.69).

## 4. Discussion

The results from this study, on the effects of extra educational elements on the confidence of undergraduate dental students learning to administer local anaesthesia, indicate a stepwise increase of confidence during the course. It is evident that students did not feel completely confident in independently administering anaesthesia to patients after the clinical training irrespective of the teaching method. Surprisingly, some students reported self-confidence prior to the first theoretical lecture. This was probably the consequence of previous professional experience working as dental hygienists or dental nurses.

The largest increase in perceived confidence was seen after the clinical exercise with students training on each other (i.e., peer training under supervision of an oral surgeon). Training with models increased the students’ confidence administering anaesthesia, but peer training in groups of three, where each student performed, received and observed the procedure, produced similar results as training on models in combination with peer training in pairs. In a full teaching schedule, it is necessary to analyse which training effort is most effective for the students. Students who were trained using anatomical models (group C) resulted in increased confidence. Training using anatomical models significantly increased students’ confidence compared to instruction using only theory. When combined with theory instruction, training on a peer increased students’ confidence even more. A survey study of 267 dental schools in Europe and Israel found that most students experienced uncertainty associated with injecting patients and desired an introduction involving anatomical models and more instruction [12]. Students positively responded to training on an anatomical model before injecting real patients.

However, studies that have investigated training primarily relying on models of jaws found no difference in the students’ ability to successfully administer local anaesthesia to a peer [5]. These findings support the idea that several teaching methods should be used in clinical training. Using models to train students in how to administer local anaesthesia does not necessarily mean that a student’s knowledge base increases [7], but such training can increase a student’s confidence and make them more comfortable for the next learning step. Wong et al. studied dental hygiene students’ experiences of anxiety related to their confidence performing local anaesthesia. An increased degree of confidence reduced students’ stress associated with administering local anaesthesia [2]. Methods other than direct clinical training have been found to be effective and improve students’ confidence administering local anaesthesia. For example, Kenny et al. found that students trained in administering local anaesthesia to children by using videos showed significantly increased confidence immediately after the training as well as at follow-up [4].

All students in the present study trained on fellow students under supervision. In group B, three students worked together—i.e., the students took turns performing, receiving and observing the procedure. In groups A and C, the students worked in pairs and administrated local anaesthesia on each other. Compared to groups A and C, group B showed significantly better results regarding student confidence administering local anaesthesia. In addition, group C, where the students practiced the procedure on one another after training on an anatomical model, showed higher confidence administering local anaesthesia than group A, where the students did not practice on an anatomical model before practicing on one another (group A). Training on a model increases students’ confidence, but training on a partner, with whom one can communicate and observe reactions, gives a better possibility of increasing self-confidence during training in local anaesthesia.

Instruction and demonstration, performed by an oral surgeon, of how to administer local anaesthesia can be compared to a master teaching a novice or a novice studying under a master. Traditionally, this has been the main pedagogical method used to train young people [13]. In medical education, much of the practical learning takes place with younger colleagues learning from older, more experienced colleagues. According to Nielsen and Kvale, observation and imitation are legitimate pedagogical methods [13]. Practical training of administering local anaesthesia can be seen as an example of legitimate peripheral participation. In the context of this study, the student observes via lectures and demonstration before administering anaesthesia on a fellow student under the supervision of a teacher. Only after practicing on a peer is the student allowed to administer local anaesthesia to a patient, also under the supervision of a teacher. According to Kary et al., the combination of these three pedagogical methods prepares students to confidently inject a patient independently of a teacher [1].

During the clinical training with local anaesthesia, students practice on each other. This arrangement, of course, raises some ethical issues. In a study on ethical issues related to the teaching of local anaesthesia at dental schools in the USA, the authors showed that the majority of training is done without oral or written consent [14]. In a survey of three dental schools in the USA, Hossaini found that many students raised ethical questions about administering anaesthesia to their peers for the sole purpose of training [15]. Furthermore, Hossaini’s generalization of the results revealed that this type of training is unethical [15]. In a survey from eleven Turkish dental colleges on how students are trained in local anaesthesia, the authors found that the clinical teaching started in the fifth semester but that the theory education started earlier [16]. The first injection was performed on peers supervised by oral surgeons. None of the schools stated that they required permission from a medical ethics committee to inject fellow students. Other dental education institutions in Sweden have similar arrangements as in Umeå, and two of the four dental schools have their students train on anatomical models before practicing local anaesthesia on a fellow student (personal communication MS). In Umeå, discussions about undergraduate training using local anaesthesia on a fellow student have been taking place, but Umeå students also expressed that they find the exercise valuable as it gives them experience with the effect of local anaesthesia. From an ethical perspective, one can ask whether receiving anesthesia in any way adds something to a student’s educational project [14]. Depending on their oral health, many young people in Sweden have never received local anesthesia during their lifetime. Gaining experience of this is important knowledge, as this experience helps students understand what their future patients will experience. The syllabus in Oral Surgery is anchored in the program council. Today, students do not give their informative consent to receive local anesthesia during the clinical training, which of course is something that should be considered in the future. However, no student is forced to receive local anesthesia if there is a contraindication, such as medical reasons or severe anxiety.

The literature describes different strategies for teaching local anaesthesia. The choice of strategies varies based on tradition, teacher availability, materials and premises and patient access. As a result of the different conditions, the literature must be evaluated based on the conclusions of each article. The readers can then apply the findings to their own institution. From the literature, we can conclude that several dental programs are working to improve the way local anaesthesia is taught. From course evaluations we know that dental students in Umeå have asked about more clinical training. Although offering more clinical training is a reasonable request, it must be incorporated into an already full training schedule. Therefore, future teaching will be conducted as before but in groups of three, with each student taking turns performing, receiving and observing the procedure. After each member of the group has performed the procedure, the clinical supervisor will guide the students in a discussion and reflection on the experience. Providing time to observe a clinical moment gives space for reflection [17]. In the situation where the student is the one administrating the anaesthetics, the focus is on the technicality of the procedure, which very likely requires the student’s full attention. The opportunity to take part in the procedure, i.e., observe the technical aspects of the injection and at the same time have the opportunity to observe the patient’s reaction, provides a greater opportunity for reflection on the overall clinical situation. This study indicates that training in groups of three improves student confidence in administering local anaesthesia compared to training only on an anatomical model.

The number of included students was limited to students who were enrolled in the course at the start of the study in spring 2020. A larger number of informants would of cause have given more strength to the study. Additionally, the students were not randomized in the different groups. At the start of the dental program, students are divided into three groups by the study administration due to technical schedule issues. All groups receive the same course content and the same amount of clinical training as previous years’ students. This means that no student is disadvantaged compared to earlier dental students.

We performed a randomization when the three groups (A, B and C) were randomized for the three test methods. The groups were not compensated for gender and age, which could have affected the results of the study, as differences in male and female dentists’ self-assessment of their clinical competence, favoring male dentists, have been reported [18]. On the other hand, the number of participants ranged between 23 and 25 and all groups had the same mean age and female dominance, although the proportion varied. In this study, we used a visual analogue scale ranging between 0–10 for evaluation of confidence. This range for the scale is often used to measure pain, but it is also used for measuring other subjective experiences, such as confidence [10,11,19]. The selected scaling method is easy to administer and easy for the students to use, and it also has good reliability and validity [20]. Additionally, the instrument has been used in other studies [10,11,21,22], which allows comparisons. A shortcoming of this measuring instrument is that the students’ self-esteem with regard to their confidence cannot always be translated into ability. The fact that the students feel safe before administrating does not mean that they have mastered it. However, it has been shown that good self-confidence and a feeling of control have a positive effect on learning [23,24].

## 5. Conclusions

The results from this study indicate that, with extra training using anatomical models before peer training in pairs, students experience a higher level of confidence when it comes to administering local anaesthesia. It was also shown that the same effect was achieved by letting students work in groups of three in the clinical situation, where each student was given the opportunity to observe and reflect on the injection procedure.

## Figures and Tables

**Table 1:** Statistical comparison between each education step using the Mann–Whitney U-test.

Comparacy between Each Education Steps	*p*-Value
Before lecture (full course, *n* = 69) vs. After lecture (full course, *n* = 69)	<0.01
After lecture (full course, *n* = 69) vs. After training in pair (Group A, *n* = 24)	<0.01
After training in pair (Group A, *n* = 24) vs. After training on model (Group C, *n* = 23)	0.01
After training in pair (Group A, *n* = 24) vs. After training 3 and 3 (Group B, *n* = 25)	0.03
After training in pair (Group A, *n* = 24) vs. After training on model and then in pair (Group C, *n* = 23)	0.02
After training 3 and 3 (Group B, *n* = 25) vs. After training on model and then in pair (Group C, *n* = 23)	0.69

**Figure A1:**
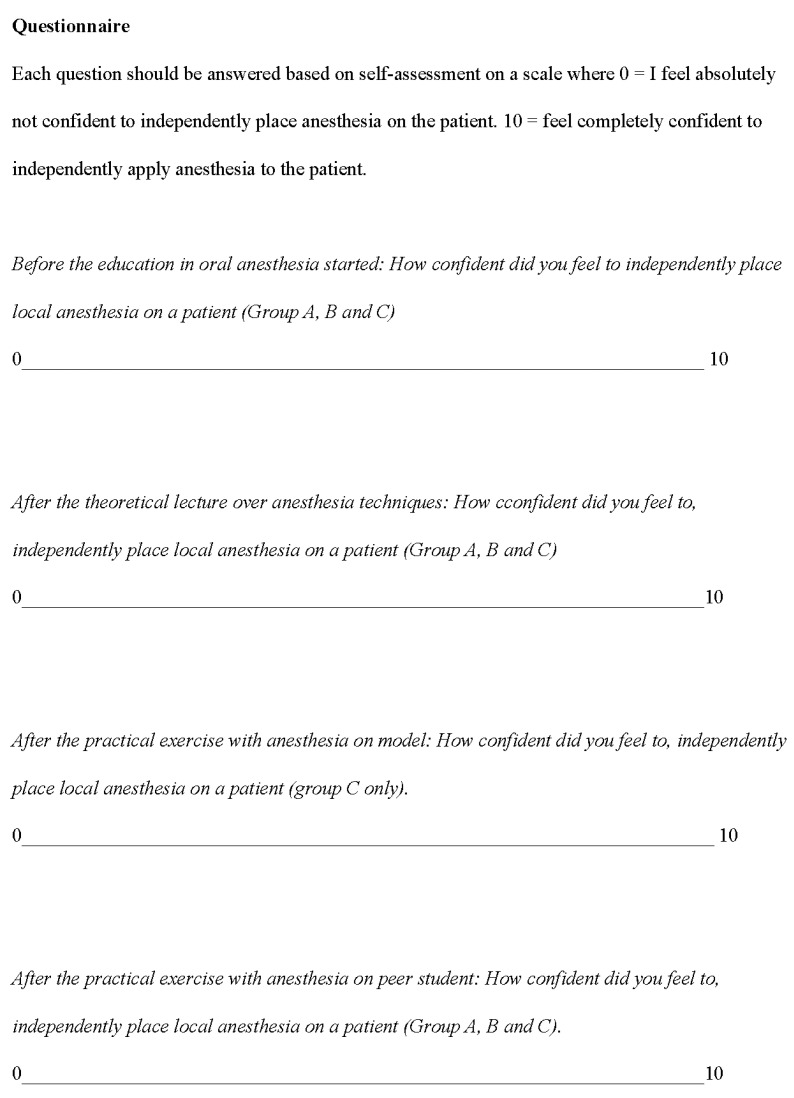
Questionnaire.

## Data Availability

The data presented in this study are available on request from the corresponding author.
